# A danish healthcare-focused economic evaluation of first-line cryoballoon ablation versus antiarrhythmic drug therapy for the treatment of paroxysmal atrial fibrillation

**DOI:** 10.1186/s12872-024-04024-5

**Published:** 2024-07-16

**Authors:** Morten Lock Hansen, Joe W. E. Moss, Jacob Tønnesen, Mette Lundsby Johansen, Malte Kuniss, Eleni Ismyrloglou, Jason Andrade, Oussama Wazni, Stuart Mealing, Alicia Sale, Daniela Afonso, Tom Bromilow, Emily Lane, Gian Battista Chierchia

**Affiliations:** 1 Copenhagen Cardiovascular Research Center, Department of Cardiology, Copenhagen University Hospital Herlev and Gentofte, Copenhagen, Denmark; 2 York Health Economics Consortium, York, UK; 3 Medtronic, Copenhagen, Denmark; 4 Kerckhoff Heart Center, Bad Nauheim, Germany; 5 Medtronic Bakken Research Center B.V, Maastricht, Netherlands; 6 University of British Columbia, Vancouver, British Columbia Canada; 7 Cleveland Clinic, Cleveland, Ohio USA; 8 Medtronic, Mounds View, Minnesota, USA; 9 Universitair Ziekenhuis Brussel and Vrije Universiteit Brussel, Brussels, Belgium

**Keywords:** Cryoablation, Atrial fibrillation, Antiarrhythmic drugs, Cost-effectiveness

## Abstract

**Introduction:**

Three randomised controlled trials (RCTs) have demonstrated that first-line cryoballoon pulmonary vein isolation decreases atrial tachycardia in patients with symptomatic paroxysmal atrial fibrillation (PAF) compared with antiarrhythmic drugs (AADs). The aim of this study was to develop a cost-effectiveness model (CEM) for first-line cryoablation compared with first-line AADs for the treatment of PAF. The model used a Danish healthcare perspective.

**Methods:**

Individual patient-level data from the Cryo-FIRST, STOP AF and EARLY-AF RCTs were used to parameterise the CEM. The model structure consisted of a hybrid decision tree (one-year time horizon) and a Markov model (40-year time horizon, with a three-month cycle length). Health-related quality of life was expressed in quality-adjusted life years (QALYs). Costs and benefits were discounted at 3% per year. Model outcomes were produced using probabilistic sensitivity analysis.

**Results:**

First-line cryoablation is dominant, meaning it results in lower costs (-€2,663) and more QALYs (0.18) when compared to first-line AADs. First-line cryoablation also has a 99.96% probability of being cost-effective, at a cost-effectiveness threshold of €23,200 per QALY gained. Regardless of initial treatment, patients were expected to receive ∼ 1.2 ablation procedures over a lifetime horizon.

**Conclusion:**

First-line cryoablation is both more effective and less costly (i.e. dominant), when compared with AADs for patients with symptomatic PAF in a Danish healthcare system.

**Supplementary Information:**

The online version contains supplementary material available at 10.1186/s12872-024-04024-5.

## Introduction

Atrial fibrillation (AF) is one of the most common types of cardiac arrhythmia worldwide, with a prevalence of around 37 million cases [1]. Symptoms commonly include light-headedness, heart palpitations and fatigue [2]. AF is also associated with an increased risk of adverse health outcomes, which include heart failure, ischemic stroke, myocardial infarction, cognitive impairment and mortality [3]. Paroxysmal AF (PAF) is an episodic variant of AF that either stops naturally or due to receiving an intervention within seven days of symptom onset [4]. If symptoms continue for more than seven days, PAF can develop into a more sustained condition, such as permanent, long-term standing persistent or, persistent AF. All of these sustained conditions increase the risk of negative cardiovascular (CV) outcomes [4].

In Denmark, Hegelund et al. (2022) estimated the prevalence of AF to be approximately 3% of the population, although they noted that the incidence had declined since 2015 [5]. A study by Johnsen et al. (2017) has also shown that AF has a substantial economic impact in Denmark, with the total 3-year attributable cost of AF, based on a societal perspective, estimated at around €219 million to €295 million [6]. Recently, three randomised controlled trials (RCTs) have investigated the use of cryoablation for initial rhythm control technique in patients who are not refractory or intolerant to AADs. The RCTs were Cryo-FIRST (NCT01803438), STOP AF FIRST (NCT03118518), and EARLY-AF (NCT02825979) [7–9]. The trials also evaluated the efficacy of cryoablation versus AADs for the prevention of atrial arrhythmia recurrence. A total of 703 patients with symptomatic PAF were randomised into two treatment arms (cryoablation and AADs). The results of the trials showed that cryoablation was superior to AADs as an initial rhythm control strategy, for the reduction of arrhythmia recurrence. Cryoablation was also associated with a low rate of procedure- or device-related serious adverse events (AEs). Furthermore, first-line cryoablation versus AADs was associated with a lower incidence of progression to persistent AF over three years [10].

This study aimed to evaluate the cost-effectiveness of first-line cryoablation versus first-line AADs for the treatment of PAF from a Danish healthcare perspective, using individual patient data (IPD), data from the Cryo-FIRST, STOP AF FIRST and EARLY-AF clinical trials.

## Methods

### Economic model structure

**Fig. 1:**
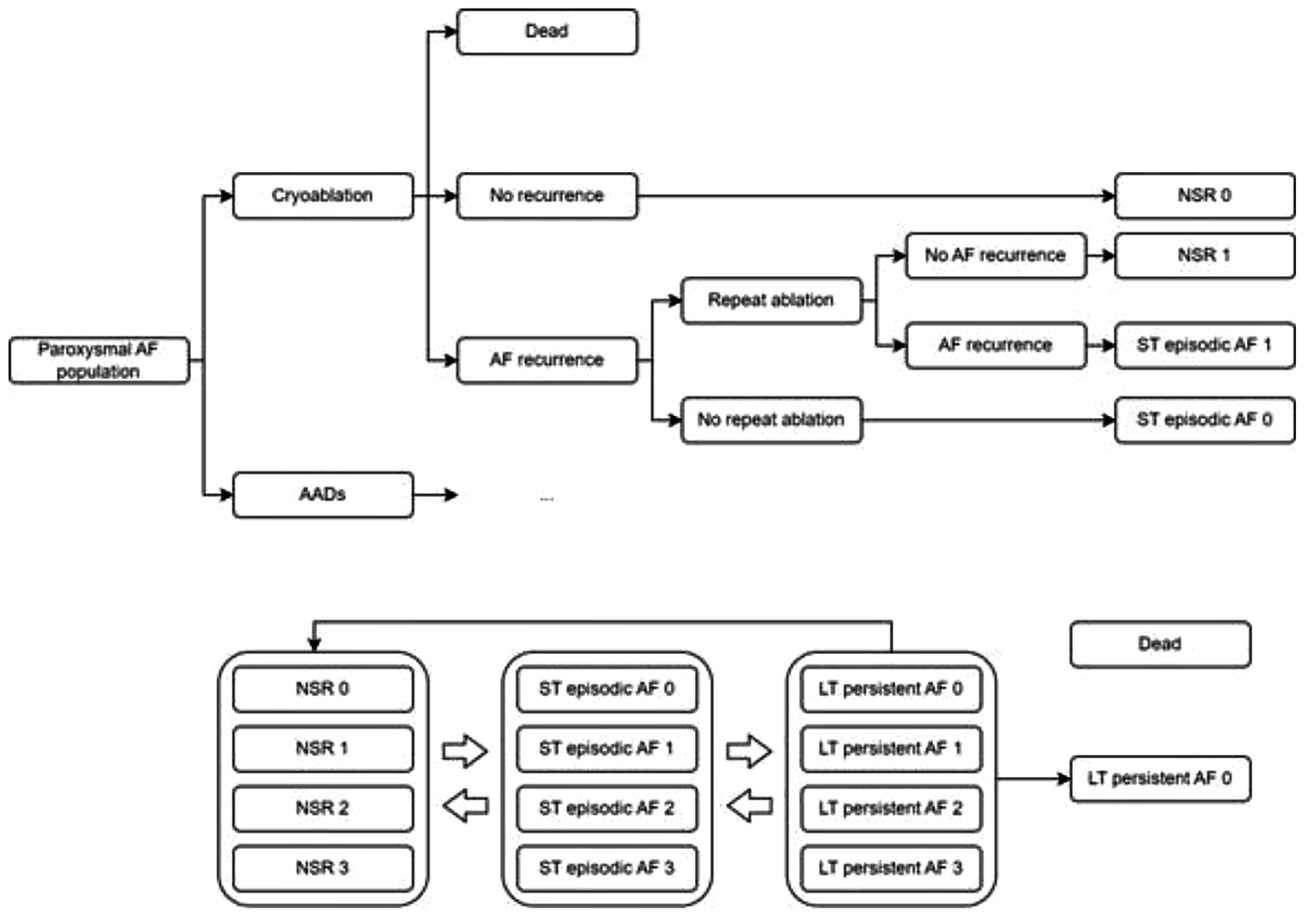
Model structure: a decision tree is used to model the initial 12 months of the economic evaluation. Each endpoint of the decision tree corresponds to a Markov model health state. The Markov model was used to model the remaining lifetime horizon. NSR, ST episodic, LT persistent AF health states are split into four sub-health states, from zero to three. This indicated the number of reablations received excluding the initial ablation in the cryoablation arm. The maximum total number of ablations in each arm was set to 3 (i.e. the cryoablation arm could not move into the “3” sub-health states). Death is an absorbing heath state. Abbreviations: AF – Atrial Fibrillation, AADs – Antiarrhythmic Drugs, ST – Short term, NSR – Normal Sinus Rhythm, LT– Long Term

An economic model using a decision-tree and Markov model structure, which was previously developed in Microsoft *Excel* for a United Kingdom (UK), Canadian and United States of America (USA) healthcare perspective, was adapted to the Danish healthcare system (Fig. 1) [11–13]. The model generated costs and benefits for a hypothetical cohort of 1,000 patients over a 40-year time horizon. A three-month cycle length was chosen to capture all the changes in AF status throughout the years, since PAF can occur at any point in time. The model patient population was based on three previous RCTs: EARLY-AF, STOP AF First, and Cryo-FIRST [7–9]. Costs and benefits were discounted at 3% per year [14].

Patients entered the economic model in the decision tree and, after 12 months, were allocated across three health states. The health states were based on clinical definitions by the European Society of Cardiology and amended to be a better fit for the economic model: normal sinus rhythm (NSR - no AF recorded within three months), short-term (ST)-episodic (at least one paroxysmal or persistent AF episode recorded within three months), and death. The health states were validated by interviewing both the clinical co-authors and external clinicians to ensure that the health states captured the disease progression and reflected clinical practice. The number of ablations following the initial procedure were also captured within the NSR and ST-episodic health states. In each arm of the model, if patients received one ablation (in addition to the initial procedure) they were allocated into the numeric sub-state ‘1’ of the health state they had previously occupied (for example, NSR-1). Patients were consequently allocated to a health state in the Markov model based on the final state in the decision tree.

The full Markov model included two additional health states: long-term (LT)-persistent, which was defined as AF symptoms that persisted over at least 12 months and did not reduce without treatments, and permanent, which was defined as AF where there were no further attempts to restore or maintain NSR. The number of ablations was also recorded in sub-health states, with patients being able to have a maximum of three ablation procedures, which included the initial procedure in the comparator arm of the model.

### Model parameters

The model inputs used in the base case analysis are shown in Table 1. Input estimates, where possible, were derived from the IPD analysis. Local clinical experts provided estimates where information was not available or collected from the literature. Where assumptions were made throughout the model, these were validated by the clinical co-authors as well as other external clinicians. A consensus was reached by all experts for the assumptions used to ensure they were reflective of their clinical experience.

**Table 1:** Key model parameters

Parameter	Value	Source
**Procedure-related costs**		
Cryoballoon	€4,554	[15]
**Healthcare contact costs per cycle**		
CV-related hospitalisations (excluding re-ablation procedures)	€ 1,996	[16]
CV-related A&E department visits (excluding re-ablation procedures)	€ 322	Costs from the UK model converted to DKK [17]
CV-related outpatient appointments (excluding re-ablation procedures)	€ 59	[16]
Pharmaceutical cardioversion	€ 1,314	Costs from the UK model converted to Euros [17]
Electrical cardioversion	€ 1,314	Costs from the UK model converted to Euros [17]
**AF-related stroke adverse events unit costs (per cycle)**		
Event costs: non-disabling stroke	€15,615	[18]
Event costs: moderately disabling stroke	€15,615	
Event costs: severely disabling stroke	€15,615	
Ongoing follow-up costs	€756	
**AF-related heart failure adverse events unit costs (per cycle)**		
Heart failure (NYHA class I)	€ 909	[19]
Heart failure (NYHA class II)	€ 909	
Heart failure (NYHA class III)	€ 909	
Heart failure (NYHA class IV)	€ 909	
**Pharmaceutical costs per arm (per cycle)**		
Cryoablation	€64	Provided by a local affiliate
AADs	€89	
**Utility decrements**		
*Health state decrements*		
LT-persistent	0.08	[20]
Permanent	0.11	
*Adverse event decrements*		
Short-term: non-disabling stroke	0.00	[21]
Short-term: moderately disabling stroke	0.37	
Short-term: severely disabling stroke	0.65	
Long-term: non-disabling stroke	0.03	
Long-term: moderately disabling stroke	0.18	
Long-term: severely disabling stroke	0.36	
Heart failure (NYHA class I)	0.00	[22]
Heart failure (NYHA class II)	0.07	
Heart failure (NYHA class III)	0.16	
Heart failure (NYHA class IV)	0.30	

*Abbreviations* AAD – Antiarrhythmic drugs, A&E – accident and emergency, CV – cardiovascular, DKK – Danish Kroner, LT – long term, NYHA – New York Heart Association

#### Statistical analysis

IPD from 703 patients with symptomatic PAF was used to inform the model parameters, such as EQ-5D utility values and the rates of outpatient appointments, pharmaceutical and electrical cardioversion, emergency department visits, AF-related hospitalisation, subsequent ablation after index treatment with cryoablation or AADs, and AF recurrence and resolution. Although similar analyses have been conducted in a UK, Canadian and USA healthcare setting, the full statistical methods, inputs and results are described in the Supplementary Materials to aid transparency for the current Danish healthcare perspective study.

Supplementary Table S1 shows the trial-specific and pooled baseline patient characteristics in the IPD analyses. If patients left the study < 30 days following the initial procedure, or < 30 days from their final ablation, they were excluded from the analysis. Each clinical trial was assigned a unique Study ID to control nesting effects in all statistical analyses, where possible. The pooled patient characteristics were assumed to represent the general first-line PAF population in Denmark. Missing data was assumed to be missing at random and the statistical analyses were conducted in R v.4.1.1 [23].

All outcomes were defined as functions of the treatment arm with further covariates of clinical relevance used to produce adjusted mean estimates. As electrocardiogram (ECG) monitoring methods differed between clinical trials, ECG method was included in all statistical models as a confounding variable. For all outcomes, generalised linear models (GLMs) and generalised linear mixed models (GLMMs) with either a Poisson (log link), Binomial (logit link), or a Beta (logit link) distribution were used. The most appropriate distribution for all statistical models was chosen based on the diagnostic criteria (e.g. Akaike’s Information Criteria) and dependent variable type (e.g. count or continuous).

An offset variable was included to derive a rate per month, rather than an absolute count, for each patient within the long-term follow-up count-based statistical models. This was done to account for exposure time for the relevant models. Utility values were obtained via the EuroQoL 5 dimensions/ 5 level (EQ-5D-5 L) instrument which was then mapped onto the 3 levels instrument (EQ-5D-3 L) by mean of the van Hout crosswalk function algorithm [24]. This was deemed appropriate for a Danish-based value set given that the crosswalk was developed to be used on international databases.

The base case model included data from all available timepoints, however a sensitivity analysis of the statistical analyses was done where the outcome data collected during a 12-week ‘blanking period’ was not taken into consideration. The ‘blanking period’ was done in agreement with the Expert Consensus Statement on Catheter and Surgical Ablation of Atrial Fibrillation, which recommends the exclusion of AF recurrences within the initial 3 months of a clinical trial [25]. The analyses described were used to assess the CEMs sensitivity to resource use within the initial 12 weeks of the clinical trial to confirm that no excessive resource use in this period affected the results disproportionally.

The full statistical analyses methods and results are shown in the Supplementary Material Table S1 to Table S17.

#### Costs and resource use

Unit costs were estimated based on diagnosis-related group (DRG) 2022 tariffs and other publicly available sources [15]. Costs that were unavailable from a Danish source, such as healthcare contact costs (see Table 1 for more detail), were converted from Great British Pounds (GBP) and Euros (EUR) to Danish Kroner (DKK) using an exchange rate of 8.73 and 7.45, respectively (as determined on 11th October 2023) [26]. However, the base case results were presented in EUR, using an exchange rate of 0.13. Cost inputs and model results reported in DKK are presented in Supplementary Material Table S18 to Table S20, respectively.

#### Utility values

Baseline utility values were based on the European EQ-5D index time trade-off (TTO) value set [27]. Disutilities based on symptom severity were estimated based on the IPD data to estimate health state-specific utility values, which were weighted by sex according to the distribution estimated from the pooled trial analysis. Disutility values for AEs were derived from the literature [21, 22].

#### Adverse events

Inputs for the AEs are reported in Table S9 to Table S14 of the Supplementary Material. The probability of stroke was estimated using the model cohort starting age, health state and the CHA_2_DS_2_-VASc score for AF stroke risk. The probability of heart failure was also estimated using the model cohort starting age, health state and general population heart failure incidence rates.

#### Mortality

General mortality was included in the model through the use of Danish IPD data which were adjusted to exclude heart failure and stroke-related deaths. Heart failure and stroke-related mortality rates [28] were combined with the rate of the respective adverse event and general mortality to calculate an all-cause mortality rate. The mortality rates were weighted by gender, using the proportion identified in the pooled clinical trial data and then converting to three months to align with the CEM. The formulae to estimate the overall mortality rates are presented in Supplementary Material 3.

#### Model outputs

The model estimates the per-patient costs and quality-adjusted life years (QALYs) for a hypothetical cohort of patients receiving first-line cryoablation compared with first-line AADs over a lifetime horizon (40 years). A cost breakdown by resource use is also estimated. The resource use included initial procedure, re-ablations, healthcare contact costs, pharmaceutical costs and AF-related AE costs. In addition to this, the time spent (in years) in each health state is also reported, as well as the total number of lifetime events and the total number of re-ablations over the lifetime horizon of the model. The cost-effectiveness ratio (ICER) and net monetary benefit (NMB) were used to measure the cost-effectiveness of first-line cryoablation compared with first-line AAD. Given that there is no official willingness-to-pay (WTP) threshold in Denmark, the approach taken was to apply the lower limit of £20,000 per QALY gained set by the National Institute for Health and Care Excellence (NICE) and convert it to and Euro equivalent, which results in an approximate WTP threshold of €23,200 [29]. This approach was previously applied in a published cost-effectiveness analysis with a Danish healthcare perspective [30].

#### Probabilistic sensitivity analysis

Probabilistic sensitivity analysis (PSA) was included in the model to quantify the impact that the uncertainty of all model parameters has on the model results across 5,000 model iterations. Mean cost and QALYs, as well as the corresponding 95% credible intervals (CrI), were reported, in addition to the ICER and the probability of cryoablation being cost-effective at a given WTP threshold. Gamma distributions were fitted to cost parameters, while beta distributions were fitted to utility and probability parameters. A Cholesky matrix was derived from the regression variance-covariance matrix to estimate the uncertainty around the statistical analysis of the IPD.

#### Scenario analysis

Scenario analyses were included in the model to explore the impact on the model results when inputs were informed by alternative sources or varied following clinical expert opinion. The following scenario analysis were taken into consideration:

1. Alternative discount rates as recommended by the Finansministeriet (Danish Ministry of Finance) [14].
2. Applying a 12-week blanking period which delayed the recording of AF recurrence within the first three months in both arms of the model.
3. Alternative utility decrements based on the European Heart Rhythm Association (EHRA) class.
4. Increasing/ decreasing the cryoablation procedure-related costs by 20%, respectively.
5. Increasing/decreasing stroke event and ongoing costs by 20%, respectively.
6. Replacing the ongoing follow-up costs of heart failure associated with each New York Heart Association (NYHA) class, which were estimated in a Danish study and were not stratified by class.
7. Increasing the relative risk (RR) of AF symptom recurrence by 10%.
8. Increasing the RR of AF symptom resolution by 10%.
9. Decreasing the probability of a successful re-ablation procedure by 30%.
10. Decreasing the incidence rate of stroke by 30%.
11. Increasing the health-state-specific stroke RR values by 10%.
12. Alternative time horizon (10 years).

## Results

### Probabilistic base case results

**Fig. 2:**
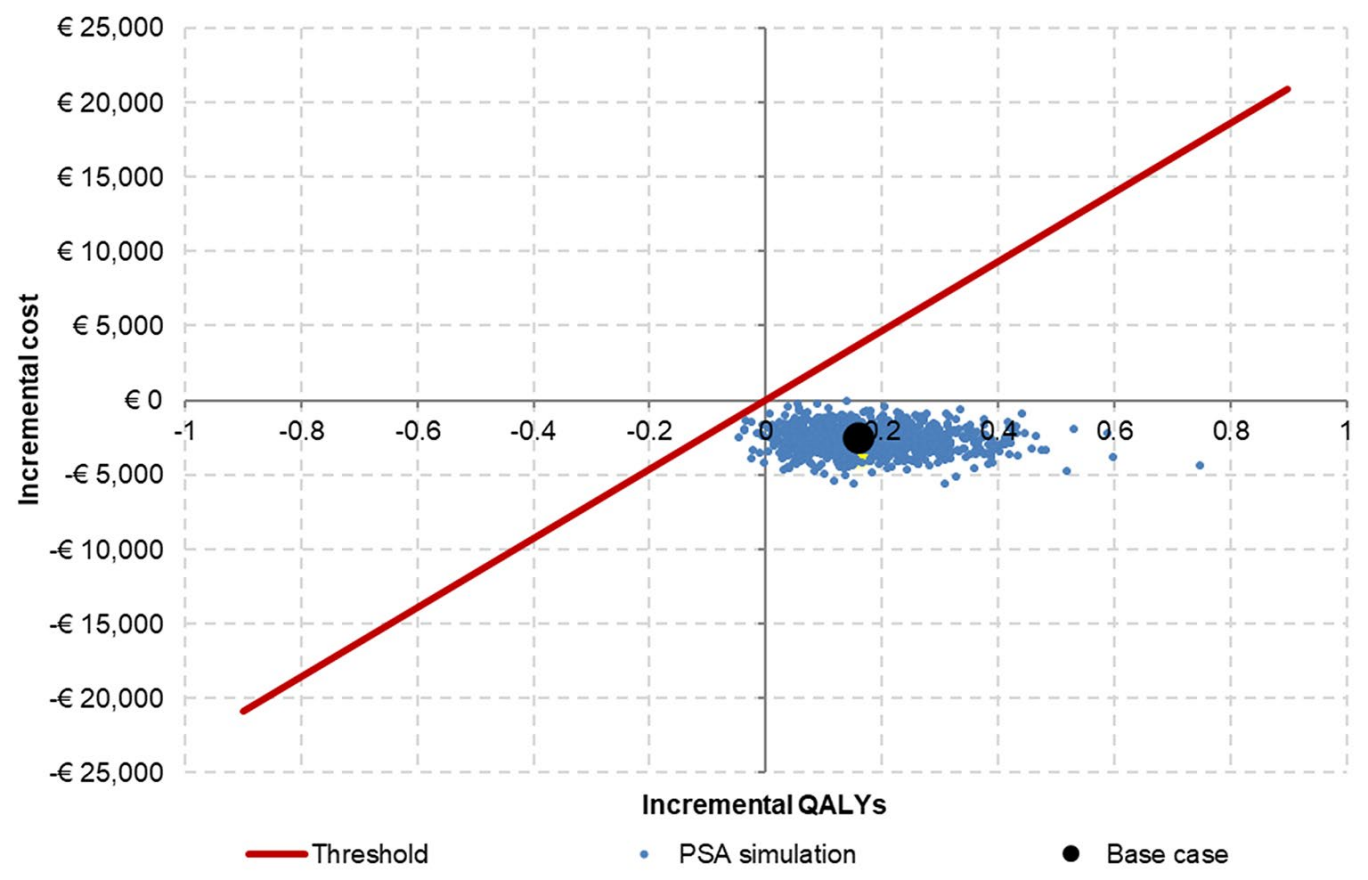
Cost-effectiveness plane: most iterations fell into the North-East quadrant below the €23,200 willingness-to-pay threshold per QALY gained. This means that first-line cryoablation is less costly and more effective than first-line AADs

As shown in Table 2, PSA results indicated that, when compared with AADs over a lifetime time horizon, the first-line cryoablation arm was estimated to generate additional QALYs (0.18; 95% Crls 0.04 to 0.28) at a lower cost (-€2,663; 95% Crls -€4,127 to -€1,255) per person (Fig. 2). First-line cryoablation was, therefore, considered dominant over AADs, with a 99.96% probability of being cost-effective at an approximate cost-effectiveness threshold of €23,200 per QALY gained.

**Table 2:** Probabilistic cost-effectiveness results (mean, 95% crls)

Outcome	Cryoablation	AADs	Incremental
Cost per patient	€23,617 [€21,497 - €25,941]	€26,280 [€24,005 - €28,822]	-€2,663 [-€4,127 - -€1,255]
QALYs per patient	13.57 [13.30–13.82]	13.39 [12.96–13.74]	0.18 [0.04–0.28]
Incremental cost-effectiveness ratio			**Dominant[€1**,**751 - €4**,**562]**
Net monetary benefit			**€6**,**871[€3**,**051 - €11**,**914]**
Probability of cost-effectiveness at a WTP threshold of €23, 200 per QALY gained			**99.96%**

*Abbreviations* AAD – Antiarrhythmic drugs, QALY – quality-adjusted life year, WTP – willingness-to-pay

Table 3 presents a cost breakdown of the deterministic results. First-line cryoablation was €4,554 more costly than AADs in terms of the initial procedure. However, this is outweighed by €7,073 in total savings in re-ablations, healthcare contact costs, pharmaceutical costs and AF-related AEs.

**Table 3:** Deterministic cost-effectiveness results (per patient)

Outcome	Cryoablation	AADs	Incremental
Initial procedure	€ 4,554	€ 0	€ 4,554
Re-ablations	€ 939	€ 4,002	-€ 3,063
Healthcare contact costs	€ 4,497	€ 6,484	-€ 1,987
Pharmaceutical costs	€ 4,383	€ 6,137	-€ 1,754
AF-related adverse events	€ 9,017	€ 9,287	-€ 270
Total cost	€ 23,390	€ 25,909	-€ 2,519
QALYs	13.59	13.43	0.16
**Incremental cost-effectiveness ratio (ICER)**			**Dominant**

*Abbreviations* AAD – antiarrhythmic drugs, AF – atrial fibrillation, QALY – quality-adjusted life year

Compared to AAD patients, cryoablation patients spent 2.12 more years in the NSR health state, and 2.10 fewer years in the short-term, long-term and permanent AF health states. The cryoablation arm also incurred 72.5% fewer ablations over a 12-month period and 76.6% fewer ablations over the whole time horizon (excluding the initial ablation procedure). Results showed that the cost per event avoided for stroke over the model time horizon is €185,000, with 73 patients needing to receive cryoablation to avoid one additional stroke. In addition, there was little difference in the lifetime heart failure rate per person between both arms of the model (-<0.001). These results are presented in Table 4. Results presented in DKK are shown in Supplementary Table S19 and S20.

**Table 4:** Additional cost-effectiveness results (per patient)

Parameter	Cryoablation	AADs	Incremental	Cost per event avoided	NNT
**Time spent in each health state (years)**					
Normal sinus rhythm	22.50	20.38	2.12		
Short-term episodic AF	2.30	3.87	-1.57		
Long-term episodic AF	0.34	0.63	-0.29		
Permanent AF	0.27	0.50	-0.23		
**Lifetime adverse event rates**					
Stroke	0.267	0.281	-0.014	€ 185,000	73
Heart failure	0.105	0.105	< 0.001	-€ 10,845,698	-4,305
**Number of re-ablations (excluding index ablation in the cryoablation arm)**					
12 months	0.07	0.25	-0.18		
Time horizon (40 years)	0.28	1.20	-0.92		

*Abbreviations* AAD – antiarrhythmic drugs, AF – atrial fibrillation, NNT – number needed to treat

### Scenario analysis results

First-line cryoablation remained dominant compared to AADs in 14 of the 15 scenarios conducted, generating less costs and more QALYs per patient (Table 5). In a scenario where the time horizon was shortened to 10 years, first-line cryoablation is still cost-effective versus AADs, although not dominant with an ICER of €13,835.

**Table 5:** Deterministic scenario analysis results (per patient)

Scenario	Incremental costs*	Incremental QALYs	ICER
Deterministic base case	-€ 2,519	0.159	Dominant
Discount rate 2.5%	-€ 2,921	0.171	Dominant
Discount rate 3.5%	-€ 2,153	0.148	Dominant
Blanking period included	-€ 3,638	0.087	Dominant
EHRA class-based decrements	-€ 2,519	0.168	Dominant
Cryoablation procedure-related costs increased by 20%	-€2,221	0.159	Dominant
Cryoablation procedure-related costs decreased by 20%	-€2,818	0.159	Dominant
Stroke event and ongoing costs increased by 20%	-€2,574	0.159	Dominant
Stroke event and ongoing costs decreased by 20%	-€2,465	0.159	Dominant
UK-based heart failure costs (converted to Euros)	-€5,522	0.159	Dominant
Increase RR of symptom recurrence by 10%	-€2,714	0.175	Dominant
Increase RR of symptom resolution by 10%	-€2,363	0.148	Dominant
Decreased the probability of successful re-ablation by 30%	-€2,570	0.166	Dominant
Decrease the incidence rate of stroke by 30%	-€2,471	0.155	Dominant
Increase health-state-specific stroke RR by 10%	-€2,535	0.161	Dominant
Time horizon 10 years	€ 818	0.059	€ 13,835

^* results for the scenario analysis have only been reported in Euros^

*Abbreviations* EHRA – European Heart Rhythm Association, ICER – incremental cost-effectiveness ratio, QALY – quality-adjusted life year, RR – relative risk

## Discussion

This study estimated the cost-effectiveness of first-line cryoablation, compared with first-line AADs, for the treatment of symptomatic AF from the perspective of the Danish healthcare system. The model results showed that first-line cryoablation was cost-effective, dominant, generating less costs and more QALYs, over a lifetime horizon. Similarly, these results were consistent with the scenarios analysis included in the model results, with first-line cryoablation remaining cost-effective over first-line AADs in all scenarios, and dominant in 15 out of the 16 scenarios assessed. First-line cryoablation also had a 99.96% probability of being cost-effective when compared to first-line AADs. This suggests that the model results are robust regardless of input uncertainty.

The main contributor to first-line cryoablation being cost-saving when compared with first-line AADs was the lower number of ablations after the index treatment over a 12- and 40-month time horizon (72.5% and 76.5% respectively). This leads to total cost savings of €3,063 per patient. Furthermore, patients in the cryoablation arm spent an additional 2.19 years in NSR health state which also contributed to a saving in healthcare contact costs (-€1,987) and AF-related AEs (-€270) per person, as well as leading to 0.16 more QALYs per person.

As mentioned previously, adaptions of the model for a UK, Canadian and USA healthcare perspective have been published [11–13]. The model results from a UK healthcare perspective showed that first-line cryoablation was cost-effective with an 89.5% probability of being cost-effective at a threshold of £20,000 per QALY gained. This model found that first-line cryoablation was cost-incurring (+£641) while still generating more QALYs (+ 0.17) when compared to first-line AADs over a lifetime horizon. Similar to our study findings, from a Canadian perspective, first-line cryoablation was dominant, with cost-savings (-CA$3,862) and higher effectiveness (+ 0.19 QALYs), when compared to first-line AADs over a lifetime horizon. This also showed that first-line cryoablation has a 99.9% probability of being cost-effective at a threshold of US$50,000 per QALY gained. First-line cryoablation was also found to be cost-effective from a USA healthcare perspective, generating more costs (US$4,274) but also more QALYs (+ 0.17), with a 76.3% probability of being cost-effective at a threshold of US$50,000. Therefore our results for the Danish healthcare perspective align with economic evaluations of cryoablation in other global healthcare systems.

In addition to the three studies mentioned earlier which used the same model structure and IPD data, other studies have been published on the cost-effectiveness of cryoablation. A study by Rodger et al. (2008) demonstrated that the lifetime costs associated with stroke risk were £14,415 for those in the cryoablation arm compared with £18,106 for those in the AAD arm [31]. These results were similar to those reported in an economic evaluation by NICE (2021) that compared second-line cryoablation to AADs and demonstrated that cryoablation was cost-effective when compared to AADs (with an ICER of £11,687 per QALY) [32].

Other cost-effectiveness studies have also been published on first-line radiofrequency ablation (RFA) compared to first-line AADs. Leung et al. (2022) showed that a significant reduction in AF recurrence and CV-related AEs results in more incremental QALYs, which resulted in an ICER of £8,614, even though there was a higher initial cost associated with the ablation [33]. However, this study only took into consideration one repeat ablation, while the current study has one initial procedure and a maximum of two repeat procedures. These studies show that cryoablation is likely to be cost-effective in multiple types of health care systems globally and, therefore, it is expected that cryoablation would also be cost-effective in a Danish healthcare setting.

A key strength of this analysis is the use of IPD analysis from three RCTs which were used to parameterise the model, where possible. Additionally, the implementation of a PSA and scenario analysis shows that the results were robust across all inputs included in the model, regardless of any assumptions used to populate some model parameters. Lastly, the model structure, parameters and assumptions were all also clinically validated.

The main limitation associated with this study is the methods used to estimate symptomatic and asymptomatic PAF events in the RCT. As a result, the re-treatment costs incurred may be overestimated due to the intensity of rhythm monitoring. However, given that the monitoring procedures were applied within the RCT it has been assumed that both arms of the model would be equally impacted by asymptomatic arrhythmia events. Published literature has shown no differences in major clinical outcomes for patients with asymptomatic versus symptomatic AF [34–36]. Given that the current study is an economic evaluation, an asymptomatic patient would not incur treatment for AF until the condition is present. Hence, they would not incur any additional treatment costs when compared with symptomatic patients. Therefore, their inclusion is unlikely to alter the conclusions of this study. Another limitation was the fact that the method for EGC monitoring also varied between the RCTs. In order to take into consideration the impact this has on the results, the statistical analysis of the IPD data included the method for ECG monitoring as a control variable [35].

The statistical analysis of the IPD data from the RCTs was used to generate input parameters. Nevertheless, due to the lack of data in the published literature, the RR parameters for AF recurrence and resolution, stroke, heart failure and re-ablation success given the number of ablations received and the health state occupied, were all based on assumptions. However, these assumptions were conservative and clinically validated. Despite these assumptions, the PSA and scenario analysis conducted showed that the model results were robust across scenarios included in the model. In addition to this, clinical expert option was taken into consideration to generate the stroke rate inputs, as well as the assumption that the utility decrements applied to the LT-persistent and ST-episodic states were equivalent.

## Conclusion

The results from this cost-effectiveness analysis suggest that first-line cryoablation is estimated to be both more effective and less costly (i.e. dominant) when compared to AADs in patients with symptomatic PAF from a Danish healthcare perspective.

### Electronic supplementary material

Below is the link to the electronic supplementary material.

Supplementary Material 1

## Data Availability

The data analysed during the current study are not publicly available due to privacy of the individuals that participated in the study. Please contact eleni.ismyrloglou@medtronic.com for questions or requests.

## Abbreviations

AAD: Antiarrhythmic Drugs
AE: Adverse Events
AF: Atrial Fibrillation
CEM: Cost-Effectiveness Model
CV: Cardiovascular
DKK: Danish Kroner
DRG: Diagnosis-related group
ECG: Electrocardiogram
EHRA: European Heart Rhythm Association
EUR: Euros
GBP: Great British Pounds
ICER: Cost-Effectiveness Ratio
IPD: Individual Patient Data
LT: Long-Term
NICE: National Institute for Health and Care Excellence
NMB: Net Monetary Benefit
NNT: Number Needed to Treat
NSR: Normal Sinus Rhythm
NYHA: New York Heart Association
PAF: Paroxysmal Atrial Fibrillation
PSA: Probabilistic Sensitivity Analysis
QALY: Quality-Adjusted Life Years
RCT: Randomised Controlled Trials
RFA: Radiofrequency Ablation
RR: Relative Risk
TTO: Time-Trade Off
UK: United Kingdom
USA: United States of America
WTP: Willingness-To-Pay
