# Supplementary material for: A danish healthcare-focused economic evaluation of first-line cryoballoon ablation versus antiarrhythmic drug therapy for the treatment of paroxysmal atrial fibrillation
Source: BMC Cardiovasc Disord. 2024 Jul 16;24:363. doi: 10.1186/s12872-024-04024-5 (PMC11251117; doi:10.1186/s12872-024-04024-5)
Supplement: Supplementary file 1 — Supplementary Material 1 [file 12872_2024_4024_MOESM1_ESM.docx]

**SUPPLEMENTARY MATERIAL**

## **Trial-specific and pooled baseline patient characteristics for the statistical analysis outputs**

**S. 1. Baseline patient characteristics from the clinical trial**

| Parameter | **STOP AF First** | | **Cryo-FIRST** | | **EARLY-AF** | | **Pooled** | |
| --- | --- | --- | --- | --- | --- | --- | --- | --- |
|  | *Cryo* | *AAD* | *Cryo* | *AAD* | *Cryo* | *AAD* | *Cryo* | *AAD* |
| Patient counts | 103 | 97 | 97 | 105 | 154 | 147 | 354 | 349 |
| Age (years) | 60.5 (11.2) | 61.3 (11.2) | 49.9 (12.6) | 54.4 (13.5) | 57.8 (11.5) | 59.7 (10.5) | 56.5 (12.4) | 58.5 (12.0) |
| Sex (% Male) | 61.17% | 58.76% | 70.10% | 64.76% | 72.72% | 69.39% | 68.60% | 65.00% |
| EQ-5D-3L derived utility | 0.89 (0.19) | 0.90 (0.15) |  |  | 0.87 (0.16) | 0.87 (0.17) | 0.88 (0.17) | 0.88 (0.16) |
| *EHRA class* | | | | | | | | |
| I |  |  | 0% | 0% |  |  |  |  |
| II |  |  | 69.1% | 75.2% |  |  |  |  |
| III |  |  | 28.9% | 23.8% |  |  |  |  |
| IV |  |  | 2.06% | 0.6% |  |  |  |  |

Abbreviations: EHRA – European Heart Rhythm Association.

***Rate of AF recurrence and resolution***

A generalised linear model (GLM) with a Poisson distribution and log-link function was used to generate the rate of atrial fibrillation (AF) recurrence and resolution. A 3-monthly rate was derived using an offset variable for the natural log of exposure time. Patients receiving cryoablation have, on average, a 46.7% lower 3-monthly AF recurrence rate than those receiving antiarrhythmic drugs (AADs). Yet, there was no statistically significant treatment impact on AF resolution (0>0.05), therefore a stepwise deletion was used to remove this from the regression model.

**S. 2. 3-monthly rate of AF recurrence (whole study duration)**

| Parameter | Coefficient | Standard Error | z-value | p-value |
| --- | --- | --- | --- | --- |
| Intercept | -2.771 | 0.175 | -15.802 | <0.001* |
| Treatment (Cryo) | -0.629 | 0.130 | -4.843 | <0.001* |
| Ambulatory device (Yes) | 0.484 | 0.216 | 2.245 | 0.024* |
| Implantable loop recorder (Yes) | 1.162 | 0.186 | 6.234 | <0.001* |
| *p* * = output reached statistical significance at a 95% confidence interval. | | | | |

**S. 3. 3-month rate of AF resolution (whole study duration)**

| Parameter | Coefficient | Standard Error | z-value | p-value |
| --- | --- | --- | --- | --- |
| Intercept | -0.441 | 0.094 | -4.665 | <0.001* |
| Implantable loop recorder (Yes) | 0.403 | 0.124 | 3.259 | 0.001* |
| *p* * = output reached statistical significance at a 95% confidence interval. | | | | |

***Rate of re-ablation***

The rate of ablation was estimated using a GLM with a Poisson distribution and log-link function. A monthly rate was estimated using an offset variable for the natural log of exposure time. A significant treatment effect was reported for the monthly rate of re-ablation (p<0.001) (Table S. *4*). Those who received cryoablation experienced an average rate of re-ablation that is 72.5% lower than those receiving AADs within the Danish cost-effectiveness model (CEM) over 12 months.

**S. 4. Rate of re-ablation (whole study duration)**

| Parameter | Coefficient | Standard Error | z-value | p-value |
| --- | --- | --- | --- | --- |
| Intercept | -3.843 | 0.108 | -35.639 | <0.001* |
| Treatment (Cryo) | -1.302 | 0.231 | -5.640 | <0.001* |
| *p* * = output reached statistical significance at a 95% confidence interval. | | | | |

***EQ-5D-3L utility inputs***

A generalised linear mixed model (GLMM) with a Beta distribution and a logit link function was used to estimate utility values (Table S. *5*). A random effect was included to control for variation between patients. After the stepwise selection, utility at baseline (p<0.001) and the treatment arm (p=0.025) were maintained as the only statistically significant predictors of utility at 12 months. Patients with short term (ST)-episodic AF in the normal sinus rhythm (NSR) health state were not found to be significantly different (p=0.115).

**S. 5. 12-month EQ-5D-3L utility inputs**

| Parameter | Estimate | Standard error | z-value | *p*-value |
| --- | --- | --- | --- | --- |
| Intercept | -0.282 | 0.260 | -1.084 | 0.278 |
| AF status (ST-AF) | -0.747 | 0.474 | -1.576 | 0.115 |
| Treatment (Cryo) | 0.219 | 0.098 | 2.234 | 0.025* |
| Baseline utility | 2.689 | 0.289 | 9.319 | <0.001* |
| *p* * = output reached statistical significance at a 95% confidence interval. | | | | |

Abbreviations: AF – atrial fibrillation, ST – short term.

**S. 6. EHRA class-based decrements**

| **Utility decrements** | **Utility decrements** | **Source** |
| --- | --- | --- |
| Heart failure (NYHA class I) | 0.00 | [1] |
| Heart failure (NYHA class II) | 0.07 |  |
| Heart failure (NYHA class III) | 0.16 |  |
| Heart failure (NYHA class IV) | 0.30 |  |

Abbreviations: NYHA – New York Heart Association.

***Cardiovascular-related hospitalization, accident and emergency visits, pharmaceutical and electrical cardioversion***

A GLMM with a Poisson distribution and a log-link function was used to derive the rates of cardiovascular-related hospitalization, accident and emergency visits, pharmaceutical and electrical cardioversion (Table S. 7). A monthly rate was estimated using an offset variable for time and a random effect was also included to control for variation between patients. A statistically significant treatment effect was found for the monthly rate of electrical (p=0.021) and pharmaceutical cardioversion (p<0.001). In addition, patients receiving cryoablation reported a 48.9% and 82.5% average reduction for a monthly rate of electrical and pharmaceutical cardioversion.

**S. 7. Cardiovascular-related hospitalization, accident and emergency visits, pharmaceutical and electrical cardioversion inputs**

| Parameter | Coefficient | Standard Error | z-value | p-value |
| --- | --- | --- | --- | --- |
| *Rate of cardiovascular-related hospitalization (whole study period)* | | | | |
| Intercept | -9.235 | 0.694 | -13.307 | <0.001* |
| *Rate of cardiovascular-related accident and emergency visits (whole study period)* | | | | |
| Intercept | -2.978 | 0.283 | -10.519 | <0.001* |
| *Rate of electrical cardioversion (whole study period)* | | | | |
| Intercept | -4.815 | 0.171 | -28.074 | <0.001* |
| Treatment (Cryo) | -0.672 | 0.291 | -2.304 | 0.021* |
| *Rate of pharmaceutical cardioversion (whole study period)* | | | | |
| Intercept | -4.234 | 0.864 | -4.898 | <0.001* |
| Treatment (Cryo) | -1.744 | 0.489 | -3.566 | <0.001* |
| Age | -0.036 | 0.013 | -2.852 | 0.004* |
| 7-day Holter | 1.574 | 0.538 | 2.923 | 0.003* |
| *p* * = output reached statistical significance at a 95% confidence interval. | | | | |

*Rate of outpatient visits*

A GLM with a Poisson distribution and a log-link function was used to estimate the rate of cardiovascular-related outpatient appointments. A random effect was included to control for variation between patients. A monthly rate was estimated using an offset variable for time.

**S. 8. Rate of cardiovascular-related outpatient appointments (whole study duration)**

| Parameter | Estimate | Standard error | z-value | p-value |
| --- | --- | --- | --- | --- |
| Intercept | -9.143 | 0.650 | -14.065 | <0.001* |
| *p* * = output reached statistical significance at a 95% confidence interval. | | | | |

## **Adverse event inputs**

**S. 9. Stroke risk by CHA₂DS₂-VASc score**

| Score | Risk | Source |
| --- | --- | --- |
| 0 | 0.2% | [2] |
| 1 | 0.6% |  |
| 2 | 2.2% |  |
| 3 | 3.2% |  |
| 4 | 4.8% |  |
| 5 | 7.2% |  |
| 6 | 9.7% |  |
| 7 | 11.2% |  |
| 8 | 10.8% |  |
| 9 | 12.2% |  |

**S. 10. CHA₂DS₂-VASc score by age**

| Age category | Score | Source |
| --- | --- | --- |
| 15 to 39 | 1.3 | Baseline study data.  Patients aged 60 - 79 have their CHA₂DS₂-VASc score increased by 1.  Those aged 80+ have their CHA₂DS₂-VASc score increased by 2. |
| 40 to 49 | 1.3 |  |
| 50 to 59 | 1.3 |  |
| 60 to 69 | 2.3 |  |
| 70 to 79 | 2.3 |  |
| 80 to 89 | 3.3 |  |
| 90 and over | 3.3 |  |

**S. 11. Stroke incidence by age and CHA₂DS₂-VASc score**

| Age category | Deterministic |
| --- | --- |
| 15 to 39 | 1.1% |
| 40 to 49 | 1.1% |
| 50 to 59 | 1.1% |
| 60 to 69 | 2.5% |
| 70 to 79 | 2.5% |
| 80 to 89 | 3.7% |
| 90 and over | 3.7% |

**S. 12. Treatment and health state-specific stroke relative risk values**

| Health state | AADs | Cryoablation | Source |
| --- | --- | --- | --- |
| NSR versus general population | 0.34 | 0.34 | Clinical assumption |
| ST-Episodic versus general population | 0.40 | 0.40 |  |
| LT-Persistent versus general population | 0.60 | 0.60 |  |
| Permanent versus general population | 1.50 | 1.50 |  |

Abbreviations: LT – long term, NSR – normal sinus rhythm, ST – short term.

**S. 13. Heart failure incidence by age in the general population**

| Age category | Deterministic | Source |
| --- | --- | --- |
| 15 to 34 | 0.004% | [3] |
| 35 to 44 | 0.013% |  |
| 45 to 54 | 0.050% |  |
| 55 to 64 | 0.200% |  |
| 65 to 74 | 0.630% |  |
| 75+ | 1.640% |  |

**S. 14. Heart failure severity distribution**

| NYHA class | Share | Source |
| --- | --- | --- |
| I | 22.14% | [4] |
| II | 40.52% |  |
| III | 28.99% |  |
| IV | 8.34% |  |

Abbreviations: NHYA – New York Heart Association.

## **Mortality inputs**

The formula shown below was used to estimate the health-state-specific overall annual mortality rates (including stroke and health failure mortality).

*All-cause mortality = [(baseline annual mortality rate [excl. stroke and Heart Failure (HF)] + (baseline annual stroke mortality rate * probability of stroke) + (baseline annual HF mortality rate * probability of HF)]*

The overall mortality rates were then converted to a 3-monthly probability so that these aligned with the cycle length and that it could be applied in the model using the formula below:

*1-exp(-rate*(cycle length/12))*

**S. 15. General population mortality rates excluding stroke and heart failure**

| Age | Male | Female | Overall | Source |
| --- | --- | --- | --- | --- |
| 15 - 19 | 0.03% | 0.01% | 0.02% | Provided by a local clinical expert: based on own calculations using data from Statistics Denmark [5]; stroke ICD-10 codes (including TCI) used: I60-I64, G458, G459 and I74. Heart failure ICD-10 codes used: I42, I50, I110 and J81 |
| 20 - 24 | 0.03% | 0.01% | 0.02% |  |
| 25 - 29 | 0.03% | 0.01% | 0.02% |  |
| 30 - 34 | 0.04% | 0.02% | 0.03% |  |
| 35 - 39 | 0.05% | 0.04% | 0.05% |  |
| 40 - 44 | 0.09% | 0.06% | 0.08% |  |
| 45 - 49 | 0.14% | 0.10% | 0.13% |  |
| 50 - 54 | 0.27% | 0.18% | 0.24% |  |
| 55 - 59 | 0.47% | 0.33% | 0.42% |  |
| 60 - 64 | 0.79% | 0.54% | 0.71% |  |
| 65 - 69 | 1.22% | 0.86% | 1.10% |  |
| 70 - 74 | 1.88% | 1.34% | 1.70% |  |
| 75 - 79 | 3.01% | 2.35% | 2.79% |  |
| 80 - 84 | 5.28% | 4.23% | 4.93% |  |
| 85 - 89 | 8.59% | 7.55% | 8.24% |  |

**S. 16. Stroke mortality rates**

| Age category | Mortality rate | Source |
| --- | --- | --- |
| 18 to 24 | 2.33% | Provided by a local clinical expert: based on own calculations using data from Statistics Denmark [5]; One-year cumulative incidence of mortality: stroke ICD-10 codes (including TCI) used: I60-I64, G458, G459 and I74. |
| 25 to 34 | 3.25% |  |
| 35 to 44 | 3.97% |  |
| 45 to 54 | 5.15% |  |
| 55 to 64 | 7.94% |  |
| 65 to 74 | 12.76% |  |
| 75+ | 28.03% |  |

**S. 17. Heart-failure mortality rates**

| Age category | Mortality rate | Source |
| --- | --- | --- |
| 16 to 24 | 13.64% | Provided by a local clinical expert: based on own calculations using data from Statistics Denmark [5]; One-year cumulative incidence of mortality: Heart failure ICD-10 codes used: I42, I50, I110 and J81 |
| 25 to 34 | 6.90% |  |
| 35 to 44 | 8.59% |  |
| 45 to 54 | 8.54% |  |
| 55 to 64 | 13.78% |  |
| 65 to 74 | 23.14% |  |
| 75+ | 41.50% |  |

## **Model results and sensitivity analysis inputs**

**S. 18. Cost inputs in Danish kroner**

| **Parameter** | **Value** | **Source** |
| --- | --- | --- |
| **Procedure-related costs** | | |
| Cryoballoon | 33,945 DKK | [6] |
| **Healthcare contact costs per cycle** | | |
| CV-related hospitalisations (excluding re-ablation procedures) | 14,880 DKK | [7] |
| CV-related A&E department visits (excluding re-ablation procedures) | 2,404 DKK | Costs from the UK model converted to DKK [8] |
| CV-related outpatient appointments (excluding re-ablation procedures) | 437 DKK | [7] |
| Pharmaceutical cardioversion | 9,801 DKK | Costs from the UK model converted to DKK [8] |
| Electrical cardioversion | 9,801 DKK | Costs from the UK model converted to DKK [8] |
| **AF-related stroke adverse events unit costs (per cycle)** | | |
| Event costs: non-disabling stroke | 116,429 DKK | [9] |
| Event costs: moderately disabling stroke | 116,429 DKK |  |
| Event costs: severely disabling stroke | 116,429 DKK |  |
| Ongoing follow-up costs | 5,635 DKK |  |
| **AF-related heart failure adverse events unit costs (per cycle)** | | |
| Heart failure (NYHA class I) | 6,774 DKK | [10] |
| Heart failure (NYHA class II) | 6,774 DKK |  |
| Heart failure (NYHA class III) | 6,774 DKK |  |
| Heart failure (NYHA class IV) | 6,774 DKK |  |
| **Pharmaceutical costs per arm (per cycle)** | | |
| Cryoablation | 475 DKK | Costs of pharmaceuticals provided by a local affiliate.   Costs applied to pharmaceutical usage derived from the IPD. |
| AADs | 666 DKK |  |

Abbreviations: AAD – antiarrhythmic drugs, A&E – accident and emergency, CV – cardiovascular, DKK – Danish Kroner, NYHA – New York Heart Association.

**S. 19. Deterministic cost-effectiveness results in Danish kroner (per person)**

| **Outcome** | **Cryoablation** | **AADs** | **Incremental** |
| --- | --- | --- | --- |
| Cost per patient | 174,399 DKK | 193,184 DKK | -18,785 DKK |
| QALYs per patient | 13.59 | 13.43 | 0.159 |
| **ICER** | | | **Dominant** |
| **Net monetary benefit** | | | **46,294 DKK** |

Abbreviations: AAD – antiarrhythmic drug, DKK – Danish Kroner, ICER – incremental cost-effective ratio, QALY – quality-adjusted life year.

**S. 20. Additional deterministic cost-effectiveness results in Danish kroner (per person)**

| **Outcome** | **Cryoablation** | **AADs** | **Incremental** |
| --- | --- | --- | --- |
| Initial procedure | 33,954 DKK | 0 DKK | 33,954 DKK |
| Reablations | 7,004 DKK | 29,839 DKK | -22,836 DKK |
| Healthcare contact costs | 33,530 DKK | 48,346 DKK | -14,816 DKK |
| Pharmaceutical costs | 32,679 DKK | 45,756 DKK | -13,076 DKK |
| AF-related adverse events | 67,232 DKK | 69,243 DKK | -2,011 DKK |
| **ICER** | | | **Dominant** |

Abbreviations: AAD – antiarrhythmic drug, AF – atrial fibrillation, DKK – Danish Kroner, ICER – incremental cost-effectiveness ratio.

**REFERENCES**

1. Göhler A, Geisler BP, Manne JM, Kosiborod M, Zhang Z, Weintraub WS*, et al.* Utility Estimates for Decision–Analytic Modeling in Chronic Heart Failure—Health States Based on New York Heart Association Classes and Number of Rehospitalizations. Value in Health. 2009;12(1):185-87.

2. Friberg L, Rosenqvist M, Lip GY. Evaluation of risk stratification schemes for ischaemic stroke and bleeding in 182 678 patients with atrial fibrillation: the Swedish Atrial Fibrillation cohort study. Eur Heart J. 2012;33(12):1500-10.

3. Christiansen MN, Kober L, Weeke P, Vasan RS, Jeppesen JL, Smith JG*, et al.* Age-Specific Trends in Incidence, Mortality, and Comorbidities of Heart Failure in Denmark, 1995 to 2012. Circulation. 2017;135(13):1214-23.

4. Zhang R, Ma S, Shanahan L, Munroe J, Horn S, Speedie S. Discovering and identifying New York heart association classification from electronic health records. BMC Med Inform Decis Mak. 2018;18(Suppl 2):48.

5. Statistics Denmark. Available from: <https://www.dst.dk/en/Statistik/emner/priser-og-forbrug/forbrugerpriser>.

6. Styrelsen S-. InteraktivDRG. 2023. Available from: <https://casemix360.solutions.iqvia.com/InteractiveProd#/>.

7. Sundhedsdata. Sygdomsbyrden i Danmark— sygdommeInteraktivDRG. 2022. Available from: <https://casemix360.solutions.iqvia.com/InteractiveProd#/>.

8. NHS England. National Schedule of NHS costs 2021/22. In; 2023.

9. Marie Jakobsen, Eskild Klaussen Fredslund, Christophe Kolodziejczyk. Omkostninger ved blodprop i hjernen og blødninger blandt patienter med atrieflimren i Danmark. En registerbaseret ’cost of illness’-analyse: 2014. Available from: <https://www.vive.dk/da/udgivelser/omkostninger-ved-blodprop-i-hjernen-og-bloedninger-blandt-patienter-med-atrieflimren-i-danmark-4z6go3xq/>.

10. Bundgaard JS, Mogensen UM, Christensen S, Ploug U, Rørth R, Ibsen R*, et al.* The economic burden of heart failure in Denmark from 1998 to 2016. Eur J Heart Fail. 2019;21(12):1526-31.
